# Probabilistic spatial analysis in quantitative microscopy with uncertainty-aware cell detection using deep Bayesian regression

**DOI:** 10.1126/sciadv.abi8295

**Published:** 2022-02-04

**Authors:** Alvaro Gomariz, Tiziano Portenier, César Nombela-Arrieta, Orcun Goksel

**Affiliations:** 1 Computer-assisted Applications in Medicine, ETH Zurich, Zurich, Switzerland.; 2 Department of Medical Oncology and Hematology, University Hospital and University of Zurich, Zurich, Switzerland.; 3 Centre for Image Analysis, Department of Information Technology, Uppsala University, Uppsala, Sweden.

## Abstract

The investigation of biological systems with three-dimensional microscopy demands automatic cell identification methods that not only are accurate but also can imply the uncertainty in their predictions. The use of deep learning to regress density maps is a popular successful approach for extracting cell coordinates from local peaks in a postprocessing step, which then, however, hinders any meaningful probabilistic output. We propose a framework that can operate on large microscopy images and output probabilistic predictions (i) by integrating deep Bayesian learning for the regression of uncertainty-aware density maps, where peak detection algorithms generate cell proposals, and (ii) by learning a mapping from prediction proposals to a probabilistic space that accurately represents the chances of a successful prediction. Using these calibrated predictions, we propose a probabilistic spatial analysis with Monte Carlo sampling. We demonstrate this in a bone marrow dataset, where our proposed methods reveal spatial patterns that are otherwise undetectable.

## Teaser

A deep learning method for cell detection in microscopy enables probabilistic interpretation of spatial analyses.

## INTRODUCTION

Advances in microscopy imaging currently enable the inspection of cells within biological tissues with astonishing resolution. In particular, fluorescence microscopy offers the possibility to examine distinct cellular structures stained with carefully selected markers. Visual inspection of such fluorescence microscopy datasets in the context of large three-dimensional (3D) images of intact tissues has revealed previously unknown information on the structures and mechanisms of different biological tissues (*1*). Effective quantitative descriptions of cellular distributions in such large datasets rely on the accuracy of cell detection, which refers to the task of identifying the presence and location of cells in the images. Despite the advances in bioimage analysis suites (*2*–*4*), this task remains challenging (*5*, *6*) and is often addressed using burdensome and time-consuming manual annotations (*1*).

Cell detection has been a fundamental challenge in the spatial characterization of hematopoiesis, the process by which blood cells are continuously produced, which mostly occurs within bone marrow tissues (*7*). Hematopoiesis takes place within a structural framework or microenvironment provided by nonhematopoietic, so-called stromal cells, which critically modulate the behavior of hematopoietic progenitors through specific interactions in restricted anatomical spaces denominated niches (*8*–*10*). A constantly increasing number of stromal cell subsets have been described to date, and their accurate identification and detection within microscopy images are key in the investigation of their functional features and dynamics in health and disease. As for all complex multicellular tissues, attempts to understand spatial patterns and cellular interactions that underlie organ function in the bone marrow rely critically on the quantification of distances between cellular coordinates and to larger supracellular anatomical structures of interest, such as blood vessels or bone surfaces (*1*, *11*–*13*).

A framework for quantification of spatial distributions was proposed in (*14*), with the use of point processes (*15*–*18*). This was demonstrated for the spatial analysis of two key stromal components within bone marrow sections: the sinusoids that form the microvasculature of the bone marrow and a pool of mesenchymal cells of fibroblastic morphology termed Cxcl12-abundant reticular (CAR) cells. The proposed spatial analysis was used to confirm the presumed preferential localization of CAR cells near sinusoids, for which no quantitative evidence had existed thus far. A number of descriptive statistics were reported in (*14*) as reference spatial descriptors of this biological system. Nevertheless, that work used semiautomatic image processing techniques based on intensity thresholding, which are inherently limited in accuracy and are time-consuming by requiring user interaction. Furthermore, although the analysis in that report provided well-established statistical methods for hypothesis testing, its strength was limited in that only the variability of the results across samples was taken into account. However, such analysis does not reflect any potential errors and uncertainty from a cell detection method used or those arising from the imaging observations, which we propose to incorporate in this study.

Automatic cell detection is a widely studied topic. A group of methods uses instance segmentation algorithms [e.g., watershed (*19*–*22*)] that assign each pixel of the image a label (cell type or background) and an identifier unique to each of the cell instances.

Although this can allow for describing cell morphology, most spatial analyses only require localization and hence center coordinates alone, thereby rendering detection algorithms more suitable for this purpose. While conventional blob detection methods (*23*, *24*) are still used in biological studies because of their simplicity (*6*, *25*–*27*), supervised deep learning (DL) approaches are slowly establishing themselves as the state of the art. The advantage of detection over instance segmentation becomes even more prominent for DL methods, where the effectiveness is determined primarily by the availability of high-quality manual annotations, because cell coordinate annotations require far less effort than dense annotation of individual pixels contained within cells.

Unlike other discriminative tasks, e.g., segmentation or classification, where end-to-end DL approaches have been widely adopted (*28*, *29*), object detection implies an underlying structure among output elements, making it a set prediction problem, for which the application of typical convolutional neural networks (CNNs) solutions is hampered (*30*). As a result, several alternatives have recently been proposed (*31*–*39*), where CNNs are used for extracting informative features, which are then subsequently processed to infer object coordinates or bounding boxes. Although these approaches have achieved remarkable results in natural images such as ImageNet (*40*), they are highly dependent on postprocessing steps that hinder their implementation and usability (*41*).

If the objects to be detected are of relatively similar size and/or their exact extents are irrelevant for the subsequent analysis to be conducted (e.g., cell counting or distances to each other or to surrounding structures), then bounding boxes are not required. In these cases, the shortcomings described above can be circumvented by using CNNs to regress density maps (DMs) generated from ground truth (GT) annotations of cell locations (*42*). Cell coordinates are then detected within these DMs as peaks, for instance, with nonmaximum suppression (NMS) algorithms. Such a framework then enables the adoption of typical CNN architectures commonly used in segmentation [e.g., UNet (*28*)] for the detection of cells and other landmarks, as used in recent works (*43*–*50*). Nevertheless, DM regression with CNNs poses two major caveats: (i) Detection of local peaks introduces additional hyperparameters that must be tuned for different applications, and (ii) detection outputs have no probabilistic interpretation; hence, uncertainties in predictions cannot be known.

Calibration is the term that refers to how well a probabilistic output value indicates actual probability of prediction success. For instance, a model that identifies 100 cells, all with a probability of 0.6, is said to be perfectly calibrated if and only if 60 of these cells turn out to be correct according to some GT, while the remaining 40 are incorrect. Although typical segmentation CNNs already output values that can be interpreted as prediction probabilities, most DL approaches are known to be poorly calibrated, invalidating any such probabilistic interpretation (*51*). Bayesian approaches that have traditionally excelled at confidence calibration have been recently incorporated in common CNN architectures. These deep Bayesian learning methods have been shown to result in better calibration by taking into account two different types of uncertainties (*52*). Epistemic uncertainty is the lack of confidence of a model on its parameters, and it may be reduced by leveraging additional labeled data. For estimating epistemic uncertainty, a technique called Monte Carlo dropout has been demonstrated to be a good Bayesian approximator of Gaussian process models (*53*, *54*) and has later been incorporated into segmentation CNNs to take pixel-wise uncertainties into account while improving the quality of their predictions (*55*). This technique was inspired by dropout layers that were originally proposed as a regularization method to mitigate overfitting by randomly removing network parameters during training (*56*). Monte Carlo dropout instead uses random removal after training during the inference phase, which enables epistemic uncertainty estimation. Aleatoric uncertainty is implicit to the data and cannot be avoided. Its estimation was proposed in (*52*) by using a loss function that accounts for noise associated with observations while simultaneously predicting this as well.

Bayesian DL models on medical images have been used to improve calibration of segmentation confidence (*57*) and to predict uncertainties as a proxy to estimate segmentation quality on previously unseen data (*58*). Similar uncertainty estimate techniques have also been successful in tasks such as image restoration (*59*) or DM regression for pose estimation (*60*). The advantages of simultaneously taking both epistemic and aleatoric uncertainties into account have been studied recently in fluorescence microscopy images (*61*), indicating that uncertainty estimation is beneficial not only for superior segmentation results but also for an accurate estimation of prediction quality on previously unseen data.

In this work, we build on the aforementioned fields by designing a probabilistic DL framework for calibrated DM-based cell detection, demonstrated in the context of large 3D fluorescence microscopy images. We validate our methods on the detection of CAR cells included in the bone marrow dataset described in (*14*). In earlier work, the complex 3D morphology of bone marrow and varying intensities of fluorescence microscopy had hindered an automatic and accurate detection of such cells, which we overcome with the methods presented herein, while furthermore demonstrating probabilistic techniques for hypothesis testing.

Below, we first give a short overview of conventional CNN-based cell detection using DMs, which we later improve with a comparative analysis for different tiling strategies and design choices. Next, we present a novel strategy with the integration of deep Bayesian methods for probabilistic classification of cell proposals, which leads to a substantial improvement in the calibration of detection results. We lastly apply our cell detection method to an extended bone marrow stroma dataset for the spatial characterization of CAR cells, revising our previously reported results. This study highlights the benefits of using well-calibrated models, allowing for a probabilistic analysis that accounts for the confidence associated to each predicted cell. This leads to findings that would be overlooked by conventional deterministic analysis.

## RESULTS

### Cell detection in DMs

**Fig. 1.**
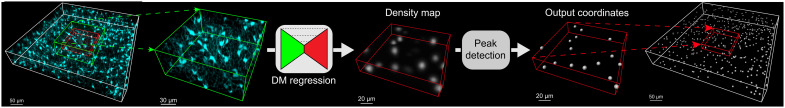
Illustration of our pipeline for cell detection. Large 3D fluorescence microscopy samples are decomposed in patches (green frame) subsequently processed by a CNN that regresses an output DM. A peak detection algorithm is applied on the DM to obtain the locations of cells. The resulting coordinates are then reconstructed within the original large sample volume based on the limits of the output patch (red frame) where they are contained.

We study the problem of cell detection on a 3D fluorescence microscopy dataset of bone marrow stroma samples, as illustrated in our overview Fig. 1. These samples are decomposed into different patches of images acquired from the bone marrow of Cxcl12–green fluorescent protein (GFP) mice, in which expression of a fluorescent protein allows for the visualization of CAR cells (see details in the “Dataset and tiling strategy” section in Methods). To quantify the quality of detection methods, an assignment is needed between predicted cell coordinates and GT annotations, such that they can be designated as true positives (TPs), false positives (FPs), or false negatives (FNs). Simple assignment strategies such as nearest neighbors may lead to bias in method evaluations, e.g., by assigning multiple detections to a single GT and vice versa—a common but undesirable scenario (*30*). We herein use the Hungarian algorithm, which finds optimal one-to-one assignments based on all relative distances between predictions and GT, as illustrated in fig. S1. This matching is applied in the context of entire samples by inverting the tiling strategy used for the creation of patches. The resulting metrics are reported following a fourfold cross-validation approach (see more details in the “Evaluation details” section in Methods).

Because DL-based regression of DMs has previously been successful in the detection of cell-like objects, we adopt this framework to localize cells as peaks in predicted DMs, whose values are related to the likelihood of occurrences. However, we need to prevent detection of multiple coordinates within a neighborhood where the typical spatial extent of a cell would physically prevent the presence of another. To this end, we use NMS to iteratively detect cells (peaks) while avoiding those closer than average cell radius (see details in the “DM generation” section in Methods). Not to detect small prediction noise as local peaks, iterative NMS process is typically terminated with a stopping criterion when no peak is left above a DM threshold, which is an empirically set hyperparameter often without further analysis in the literature (*42*); thus, we evaluate it for different DM design strategies in the next section.

### Tiling strategy and DM design for large images

**Fig. 2.**
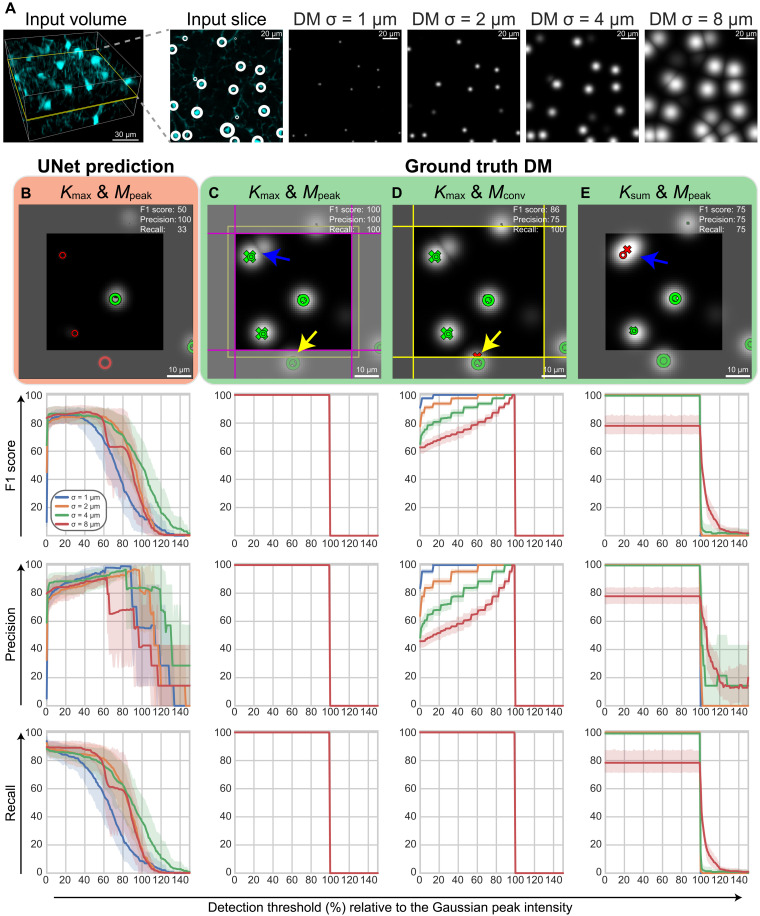
Analysis of DM design alternatives for regression-based cell detection assuming perfect GT DM predictions. (**A**) Example slice of DMs created with Gaussian kernels with different σ values. White rings in the input image denote GT out-of-plane coordinate with size proportional to proximity to the displayed slice. (**B** to **E**) Comparison of different detection algorithmic choices and thresholds for the application of NMS on GT DMs (green) and DMs predicted with UNet (red). Example DMs are shown in the top with their respective detection metrics. Predicted and GT coordinates are matched only within a given patch (bright square in the center). Yellow lines show the tiling boundary (CNN patch size) in the *M*_conv_ strategy. Magenta lines depict the patch size for the *M*_peak_ strategy. GT annotations are represented as rings (∘), and predictions are represented as crosses (×). Both ∘ and × are green when they form a TP pair or red for FP or FN. The yellow arrows mark an example where *M*_peak_ helps, and the blue arrows mark another example where *K*_max_ helps. The graphs show the detection metrics as the mean (solid line) and 95% confidence interval across samples (*n* = 7) in the test set across detection thresholds, for each tested experimental setting and σ value.

While DMs are used as a suitable framework for cell detection, their design specifics vary considerably. DMs encode cells as a local kernel with a peak at its center and monotonically decreasing outward. We specifically use a 3D Gaussian kernel with an SD σ (illustrated in Fig. 2A), the nearly flat peak of which is advantageous for learning from GT annotations that may not be precise. In this section, we propose the use of two DM design strategies. These methods are first evaluated on ideal GT DMs where detection metrics should reach perfection (i.e., 100%) and be as insensitive as possible to hyperparameter selection. In addition, this assessment allows for comparisons to remain agnostic to chosen DM prediction methods.

Tiling strategies are used to decompose large images in small patches that can be effectively processed, because their memory footprint in typical CNNs is otherwise too big to be effectively handled. Traditional tiling methods for image segmentation use input image patches larger than expected prediction output to take into account the pixels lost in convolutional layers (*62*, *63*), which we refer to as convolutional margin (*M*_conv_). In the context of detection, *M*_conv_ often leads to detection of multiple duplicated cells in neighboring patches, which need to be somehow combined, often leading to a lower detection precision (Fig. 2D). This effect is accentuated for higher values of σ and lower DM thresholds, because these factors increase the chances of localizing coordinates at patch borders, where the actual peak may lie within a neighboring patch. The *M*_peak_ tiling strategy that we propose uses a smaller output patch (magenta), which is obtained by cropping the CNN output (yellow) with a small distance to ensure that duplicated detections falling within that margin are considered only in one of the patches (yellow arrow in Fig. 2, C and D, and illustrated in 1D in fig. S2). The patches obtained with this strategy are contiguous along the magenta lines, as detailed in the “Dataset and tiling strategy” section in Methods.

The second strategy (*K*_max_) is designed to address a problem arising in the GT DM generation by using the process of kernel sum (*K*_sum_), an approach common in earlier works (*28*, *42*). Adding kernels with *K*_sum_ from close-by coordinates may erroneously result in single peaks. In addition, *K*_sum_ artificially increases the dynamic range of DMs complicating CNN predictions and confounding peak density with prediction strength (nearby peaks leading to relatively larger GT DM responses). This problem is illustrated in fig. S3 and marked with a blue arrow in an example image in Fig. 2E. With *K*_max_, Gaussians are compounded by their maximum, as recently proposed in a different experimental setting (*49*), hence facilitating their separation by interpreting densities as an intersection rather than an accumulation and eliminating the sensitivity of detection metrics to the spatial spread of a kernel, i.e., σ of Gaussian.

Careful selection of σ and detection threshold values can indeed produce perfect detection results on GT DMs with either of the traditional *M*_conv_ or *K*_sum_ strategies. However, any sensitivity to these two hyperparameters is undesirable when the detection method is to be applied on CNN predictions, which are expected to produce DM values and shapes differently to those observed for GT DMs. As described in the next section, an optimum detection threshold can be found, e.g., via grid search on a validation set once a model has been trained, but a similar approach for σ would require the training and evaluation of distinct models for each σ value, which is a cumbersome task. The observation that *K*_max_ & *M*_peak_ produce perfect detection metrics on GT DMs with any evaluated sigma and threshold between 0 and 100% suggests that this design strategy is a better candidate for detection on predicted DMs, as it can alleviate the dependency on hyperparameter values that would lead to suboptimal detection results (see details in table S1). We subsequently train a UNet model for the prediction of these DMs with the results shown in Fig. 2B, where the quality of the detections achieved with our *K*_max_ & *M*_peak_ is observed to highly depend on the detection threshold. While too small or too high thresholds are consistently worse, a high variation is observed across evaluated test samples, contrary to the clearer patterns observed for detection on GT DMs.

### Probabilistic classification of cell proposals

Application of NMS with a DM-based threshold hinders cell detection by (i) introducing a hyperparameter that, as shown in the previous section, highly influences the detection quality and (ii) preventing a probabilistic interpretation of the predictions that can be linked to their confidence. We hereby propose an alternative that addresses these drawbacks without compromising the detection quality. First, we generate numerous cell proposals by using NMS on predicted DMs without applying a threshold (effectively setting the threshold to 0), which guarantees high recall but low precision because all possible peaks are considered as cells, as shown in Fig. 2B. We fix σ to the expected cell radius of 4 μm following the guidelines proposed on related work (*28*, *42*, *43*). Our results in Fig. 2B and table S1 confirm that this value of σ provides favorable detection metrics. Second, we aggregate summary statistics from the neighborhood of each proposal as a feature vector. These features act as input to a binary classifier that, in contrast to the deterministic threshold previously used, assigns each of the proposals a probability of being a cell. Our method hence provides a mapping from the regressed DM to a probability space for each identified cell.

**Fig. 3.**
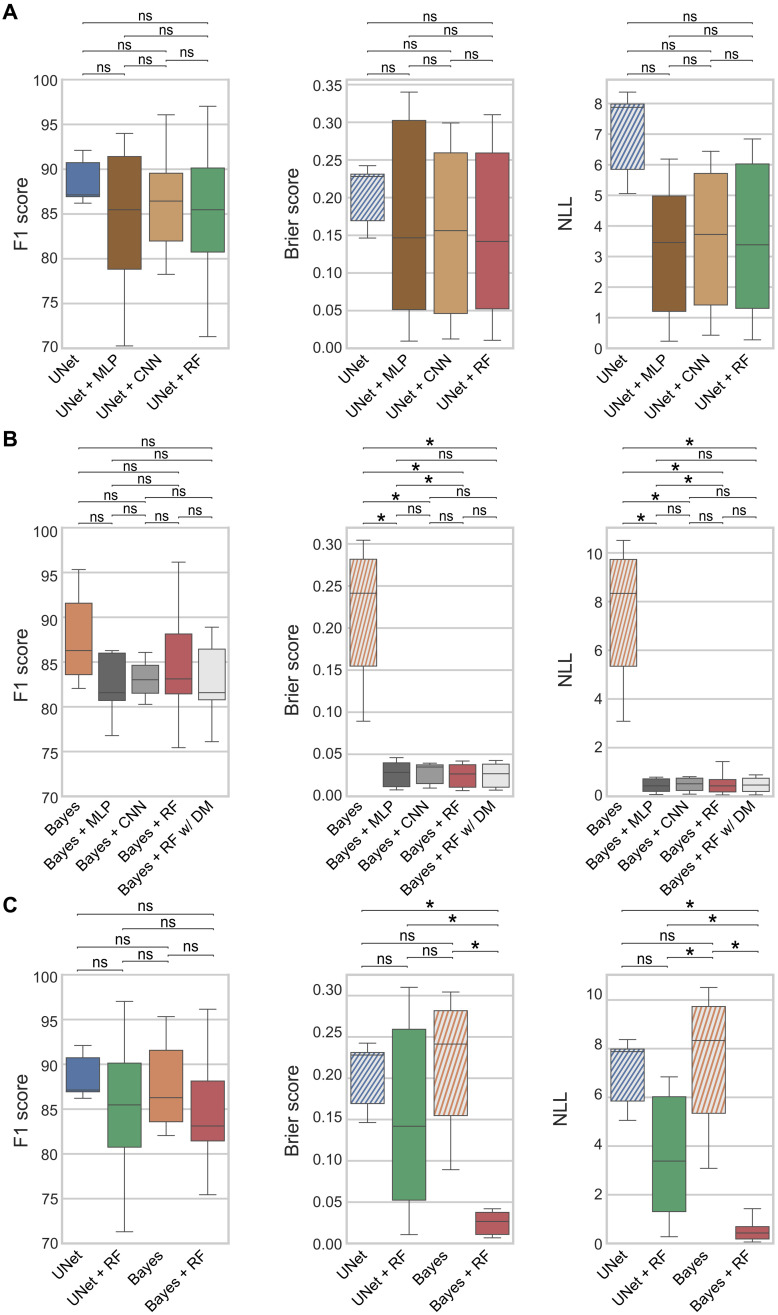
Prediction metrics for the different proposed cell detection models. (**A**) Comparison between standard UNet, for which the DM threshold is selected from the validation set, and its extension with different probabilistic classifiers that do not require defining a threshold. ns, not significant. (**B**) Comparison of different probabilistic classifier strategies added to Bayes. (**C**) Comparison between UNet and its Bayesian implementation Bayes when selecting the DM threshold from the validation set and the respective models when adding a probabilistic binary classifier RF (*+*RF). Brier score and NLL evaluate probabilistic predictions (lower is better). Because threshold-based models are deterministic, they are evaluated by assigning a fixed probability of 1 to positive predictions (TP or FP) and 0 to FN (shown in stripes to indicate this evaluation convention). All models are evaluated by fourfold cross-validation on the test set (*n* = 7). Significance is indicated with *P* value≤0.05 (*).

In Fig. 3A, we compare different classifiers added onto the above baseline DM estimator with the baseline UNet where the DM threshold is selected from a validation set (see details in the “Evaluation details” section). As classifiers, we compare a multilayer perceptron (UNet *+* MLP), a CNN (UNet *+* CNN), and a random forest (UNet *+* RF). The results show that any of these classifiers can effectively remove the need for the said detection threshold, without sacrificing detection accuracy, i.e., significantly affecting the F1 score.

Although the results for the different binary classifiers are comparable, we propose the use of UNet *+* RF, as RFs have been described to be robust against overfitting, produce well-calibrated results, and provide accurate predictions when input features contain representative descriptions of the output labels (*61*, *64*). This latter characteristic is particularly suitable for our task, as we expect our predicted DMs to display information for easy discrimination of cell instances.

Brier score (*65*) and negative log-likelihood (NLL) are scoring metrics used for the evaluation of calibration quality (lower is better) of probabilistic predictions (*51*, *66*). These scores indicate that UNet *+* RF leads to a slight yet not significant improvement in calibration over UNet.

### Bayesian regression networks for calibrated detections

Motivated by the advantages in calibration with the use of different Bayesian DL techniques reported for different domains (*52*, *57*, *58*, *61*), we adapt our framework to take into account any uncertainties in DM regression. We denote our Bayesian DL approach as Bayes, described later in the “Neural networks for DM regression” section in Methods. In addition to a predicted DM, this method also produces spatial maps of epistemic and aleatoric uncertainties, which can be provided to the classifier as additional input as described in the previous section. As seen in Fig. 3B, any of the tested classifier combination with a Bayesian network substantially improves the calibration quality of Bayes seen with Brier score and NLL. Furthermore, Fig. 3C shows that this superiority in calibration results is achieved with Bayes *+* RF over any of the other models evaluated (based on either UNet or Bayes) without differing significantly in detection quality (F1 score).

We explore whether the inference time of our Bayes model can be reduced by only estimating aleatoric uncertainty. Such Bayes aleatoric CNN requires a single forward pass (0.02 s per patch) instead of the multiple passes required for the epistemic uncertainty also considered in Bayes (2.00 s per patch). However, we show in fig. S4 that Bayes aleatoric leads to significantly worse Brier and NLL metrics.

Although we use RF because of its advantages described in the previous section, we show in Fig. 3B that replacing it with other binary classifiers, namely, an MLP or a CNN, produces similar results. Furthermore, the detection quality is barely affected upon changing different hyperparameters of the RF classifier (table S2). Omitting uncertainties and using RF only on features created from the DM (RF w/ DM) do not result in deterioration of detection quality nor calibration. This observation implies that, for Bayesian networks, the predicted DMs may alone hold most information necessary for inferring the prediction probability. However, as seen in Fig. 3C, the Bayesian output alone (Bayes) is not well calibrated, yielding even poorer Brier score and NLL than UNet *+* RF. These results indicate that the use of a binary classifier as we proposed in the previous section is indeed key to obtaining accurate confidence estimations. Comparing the DL approaches, this classifier truly excels in calibration when used on Bayesian predictions (Bayes *+* RF), where the Brier score and NLL are substantially lower than those for UNet *+* RF. Overall, the best results in detection accuracy (F1 score) and calibration (Brier score and NLL) are achieved using Bayes *+* RF on feature vectors including uncertainty statistics, a method setting that we used in all further experiments.

### Qualitative assessment of detection proposals

**Fig. 4.**
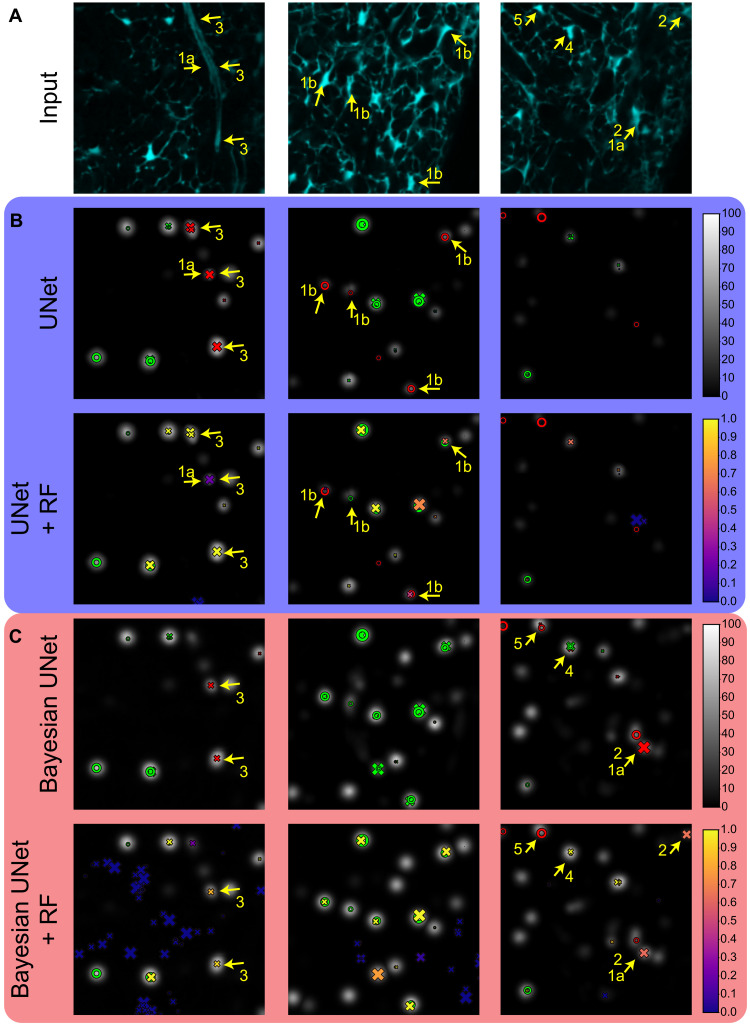
Visualization of predictions with different proposed models on example 2D slices of the test set. GT coordinates are represented by rings (∘) and predictions by crosses (×), with a size proportional to their proximity to the displayed slice, whose input image is displayed in (**A**). In deterministic models (**B**), coordinates are colored green when they have a positive match (TP) and red when they do not (FP for × and FN for ∘), according to Hungarian matching. In probabilistic models (**C**), the predictions are colored according to their associated probability (color bar at the right), whereas GT annotations follow the deterministic coloring scheme, considering positive predictions as those with *P* ≥ 0.5. Arrows mark specific examples discussed in the text according to their accompanying number.

To better understand the behavior of our presented deterministic and probabilistic models, we include in Fig. 4 visualizations of example predictions on the test set, with relevant findings marked with numbered arrows. Examples marked with arrows 1 show the benefits of probabilistic models on several predictions that are mistaken in their deterministic counterpart, either by assigning probabilities below 1 to FP (1a) or above 0 for FN (1b). While, in the best cases, these predictions are turned respectively into TN (*p* < 0.5) or TP (*p* ≥ 0.5), simply taking their probabilities into account lowers the confidence of wrong predictions, a benefit confirmed by the lower Brier score and NLL of probabilistic models (Fig. 3A).

We also report a number of observed failure cases. Some FPs marked with arrows 2 appear as TPs given the input image, and these may indeed have been mistakenly overlooked during GT annotation. All these observed examples have a relatively low prediction confidence (0.5 ≤ *p* < 0.75), potentially explaining or emulating the annotator’s oversight. FPs marked with arrows 3 are due to the presence of an artery: a structure unobserved in the training set that displays high intensities even in the absence of cells. Other errors are due to the prediction of large density blobs, either producing multiple predictions for a single GT (arrow 4) or producing a local maximum too far from its respective GT (arrow 5).

### Transferability of probabilistic cell detection to a different cell type

**Fig. 5.**
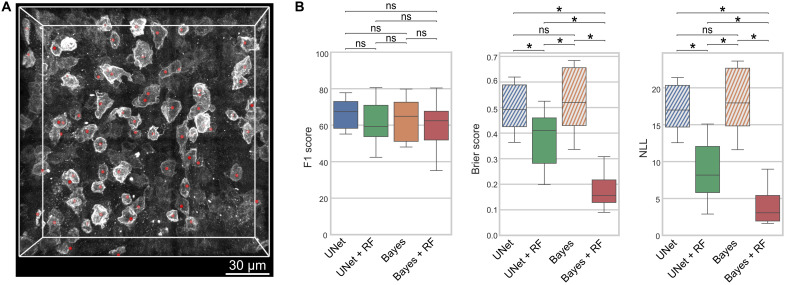
Transferability of the proposed methods to c-kit^+^ cells. (**A**) Example 3D image acquired from the c-kit marker as a grayscale maximum intensity projection and the corresponding c-kit^+^ manual annotations as red dots. (**B**) Detection (F1 score; higher is better) and calibration (Brier score and NLL; lower is better) metrics of the different methods on the c-kit^+^ cells. Threshold-based models are shown in stripes to highlight that a fixed probability of 1 is assigned to positive predictions (TP or FP) and of 0 to FN. All models are evaluated by fourfold cross-validation on the test set (*n* = 7). Significance is indicated with *P* value ≤0.05 (*).

In this section, we apply our proposed methods to a different set of cells present at higher densities in bone marrow tissues to validate the consistency of our findings. We herein use images generated by the acquisition of a c-kit staining, a marker known to identify a broad subset including hematopoietic stem and progenitor cells, from which all mature blood cells are generated (*12*, *67*). We train our models to find the manually annotated c-kit^+^ cells visualized in Fig. 5A. As detailed in table S3, c-kit^+^ annotations are 2.96 ± 1.96 times denser than those of the CAR cells used in the previous sections.

Figure 5 confirms the findings in Fig. 3 for CAR cells. While substituting the DM thresholding approach (as in UNet and Bayes) by our probabilistic cell classifier (UNet *+* RF and Bayes *+* RF) does not significantly alter the detection F1 score, it does substantially improve the calibration of the predictions. This is seen by the lower Brier score and NLL when RF is used in combination with either UNet or Bayes. The best calibration results are achieved with Bayes *+* RF.

### Probabilistic spatial characterization of bone marrow stromal cells with calibrated cell detection

**Fig. 6.**
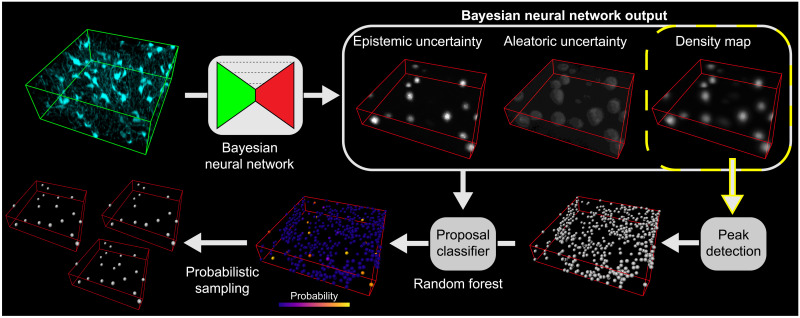
Representation of our proposed method for probabilistic cell detection. Input patches are processed by a Bayesian CNN (Bayes in the main text) to regress an output DM and its corresponding uncertainties. These outputs are used by a classifier (RF) to assign a probability or confidence to a large number of cells proposed by the application of peak detection on the DM. The resulting proposals can be sampled according to their assigned probability.

**Fig. 7.**
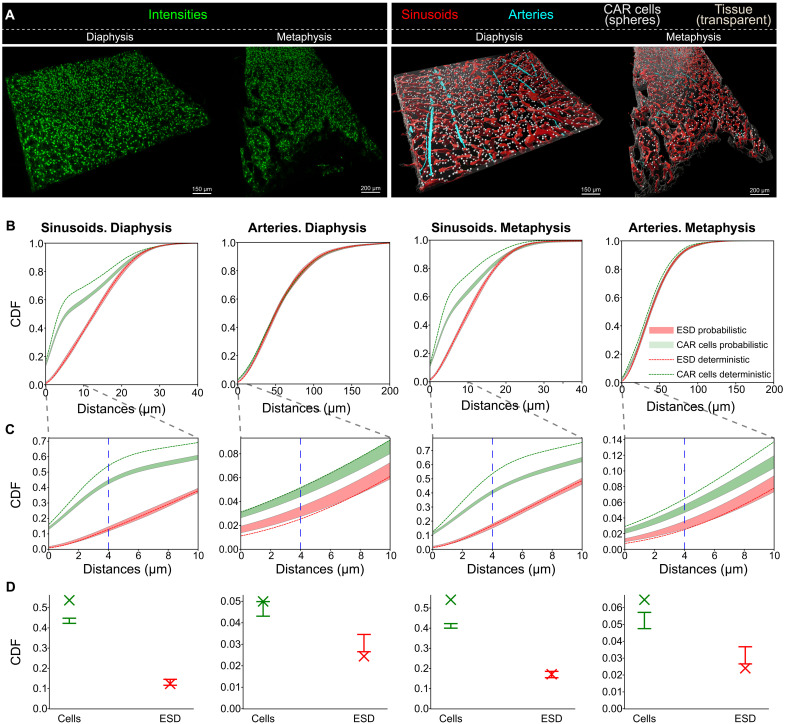
Quantification of spatial distributions in the bone marrow stroma with our methods producing calibrated cell detections. (**A**) Example visualizations of bone marrow diaphysis and metaphysis regions, with the intensities used for detection of CAR cells (top) and the computational representation (segmentation and detection) of the different structures used in the analysis (bottom). (**B**) CDF of distances to sinusoids and arteries for both bone marrow regions, zoomed-in in (**C**) to highlight the confidence ranges of the probabilistic analysis. Dashed lines are used for deterministic results. Probabilistic results are represented as envelopes containing the maximum and minimum CDF values for each distance (*n* = 50). (**D**) Cross section of the CDF at a distance of 4 μm [blue dashed line in (C)] to emphasize the differences between probabilistic measures capturing a confidence range (depicted as $\bar{⊥}$) with their deterministic counterpart that calculates a single value (depicted as ×). An extension of this figure with GT data is included in fig. S5, which confirms that the probabilistic results are closer to those achieved with GT annotations within this sample.

In this section, we deploy our calibrated cell detection framework (illustrated in Fig. 6) to revise the quantification of CAR cell spatial distributions in the context of bone marrow stroma presented in (*14*), where an analysis of densities of this cell type and its spatial associations was evaluated with deterministic conventional image processing (CIP) methods that limited the statistical power of the observations. The use of spatial point processes was proposed to describe patterns with cumulative distribution functions (CDFs) of empty space distances (ESDs) and distances from CAR cells, which we apply here (see details in the “Pipeline for quantification of bone marrow stroma” section in Methods) to describe their distribution relative to sinusoids and arteries segmented in a separate study (*62*) for diaphysis and metaphysis, two distinct bone marrow regions shown in Fig. 7A.

We propose two different types of analysis, for which the probabilities from Bayes *+* RF predictions are treated differently. A deterministic result is extracted for each bone marrow sample by analyzing only cells predicted with *p* ≥ 0.5, which contribute equally to the analysis regardless of their prediction confidence. Alternative results are extracted with a probabilistic strategy that repeats the experiment multiple times (herein 50). For each of the repetitions, the analysis is performed with a different set of cells selected by sampling the predicted cell proposal by their corresponding *p* assigned by the probabilistic classifier. For example, two cells with *p* = 0.9 and *p* = 0.02 are expected to appear, respectively, in 45 and 1 of the 50 replicates. The contribution of each cell to this probabilistic analysis hence becomes proportional to its associated confidence, which we hypothesize to statistically be more rigorous than the deterministic interpretation, as also evaluated with the experiments below. More details on both the deterministic and probabilistic strategies can be found in the “Probabilistic spatial characterization of bone marrow stromal cells with calibrated cell detection” section.

A comparison of the observations reported with CIP in (*14*) with the deterministic and probabilistic analyses hereby presented is shown in Table 1. The deterministic interpretation of the outputs achieved with Bayes *+* RF already increases the statistical power of the previous quantification with CIP, as our presented method allows for automation, applicability to more samples, and better detection metrics. Application of our probabilistic analysis reveals that, relative to the deterministic results, there is an increase in the global density of cells and a decrease of them in the vicinity of both sinusoids and arteries. The probabilistic interpretation of ESD implies random sampling of the empirical values; hence, the similarities to deterministic result in % Volume and between ESD CDFs. Although a sample-wise aggregation of the values is included for comparison with earlier works, we also aggregate them for the entire dataset as a final quantitative report emphasizing that only a probabilistic analysis accounts for the uncertainty of the predictions.

**Table 1.** Analysis results on bone marrow stroma dataset as previously reported with CIP and the deterministic and probabilistic models proposed herein. Adjacency refers to distances smaller than 4 μm. SD is measured across samples in sample-wise rows and across prediction replicates (*n* = 50) in aggregated dataset rows. All results are shown as means **±** SD. Results for CIP are taken directly from (*14*), as they constitute to date the state-of-the-art results in quantitative characterization of spatial distribution of CAR cells in bone marrow tissues. The deterministic and probabilistic parameters obtained with the DL detection methods herein proposed rely on the segmentation of sinusoids and arteries described in (*62*). The reported detection metrics have a deterministic interpretation only and are hence similar for both deterministic and probabilistic analyses. Nevertheless, the differences in the other rows are the result of the sampling process, which use the probabilistic interpretations in the analysis; cf. the large differences in cell percentages adjacent to sinusoids. The best metrics are emphasized in bold. The calibration metrics indicate that our probabilistic method much better estimates the chances of a result being accurate. More details on these metrics can be found in the “Evaluation details” section in Methods. n/a, not applicable.

	**Method**	**CIP** (*14*)		**Deterministic**		**Probabilistic**	
		**Region**	**Diaphysis**	**Metaphysis**	**Diaphysis**	**Metaphysis**	**Diaphysis**	**Metaphysis**
**Sample-wise** **analysis**	Density (cells/ mm^3^ × 10^4^)	3.78 ± 0.14	3.21 ± 0.14	3.02 ± 0.32	3.05 ± 0.47	3.30 ± 0.23	3.41 ± 0.27
	% Cells adjacent to sinusoids	64.0 ± 0.7	n/a	50.71 ± 5.78	51.46 ± 3.92	42.89 ± 4.56	43.94 ± 2.60
	% Volume adjacent to sinusoids	n/a	n/a	11.71 ± 1.15	14.46 ± 2.74	11.73 ± 1.71	14.45 ± 2.74
	% Cells adjacent to arteries	n/a	n/a	5.10 ± 4.30	3.58 ± 1.48	4.53 ± 3.29	3.14 ± 1.24
	% Volume adjacent to arteries	n/a	n/a	1.21 ± 0.65	1.19 ± 0.49	1.24 ± 0.79	1.18 ± 0.50
**Aggregated** **dataset analysis**	Density (cells/ mm^3^ ×10^4^)	n/a	n/a	3.07	2.99	3.28 ± 0.01	3.41 ± 0.01
	% Cells adjacent to sinusoids	n/a	n/a	50.23	50.78	42.97 ± 0.14	43.06 ± 0.17
	% Volume adjacent to sinusoids	n/a	n/a	14.93	11.37	11.38 ± 0.14	14.92 ± 0.23
	% Cells adjacent to arteries	n/a	n/a	4.78	4.26	4.32 ± 0.07	3.74 ± 0.11
	% Volume adjacent to arteries	n/a	n/a	1.31	1.39	1.31 ± 0.06	1.36 ± 0.11
**Throughput**	No. of samples (used for arteries)	6 (0)	6 (0)	33 (24)	10 (6)	33 (24)	10 (6)
	Time required	15 min per sample manual		**Automatic** training: 110 min; inference: 2 s per patch			
**Detection metrics**	F1 score	74.76 ± 9.63		**84.84 ± 6.73**			
	Precision	73.49 ± 21.77		**83.84 ± 9.72**			
	Recall	83.63 ± 15.38		**86.62 ± 7.73**			
**Calibration** **metrics**	Brier (%)	39.48 ± 12.56		25.81 ± 10.47		**3.46 ± 3.65**	
	NLL	13.64 ± 4.34		8.91 ± 3.62		**0.52 ± 0.48**	

The application of deterministic analysis in the characterization of distances through their CDFs, shown in Fig. 7 (B to D), confirms the previously reported trend of the preferential localization of CAR cells in close proximity to sinusoids, both in diaphysis and metaphysis. Furthermore, the access to segmented arteries unavailable in (*14*) allows us to describe a much subtler tendency of CAR cells to also be preferentially residing within the vicinity of these specialized vascular structures. The application of the probabilistic analysis permits the extraction of these curves as confidence intervals (envelopes) reflecting the confidence in results when taking into account prediction probabilities. Notably, the deterministic curves lie outside these envelopes for large spatial distance ranges. As shown in fig. S5, the probabilistic spatial analysis more accurately captures the results obtained from the same analysis on manually annotated GT cells. Together, these results suggest that a probabilistic analysis is beneficial not only in taking uncertainty into account but also in potentially revealing patterns only visible by considering confidence associated to the model’s predictions.

## DISCUSSION

In this study, we propose a DL-based cell detection framework designed for its application on 3D fluorescence microscopy datasets that addresses several problems in previous DM regression methods for detection. In addition, we introduce a method for well-calibrated probabilistic predictions that can be incorporated in powerful statistical analysis frameworks.

We first argue the importance of adapting existing tiling strategies to take a supplementary margin into account (*M*_peak_), without which any detection results are inherently flawed, even upon perfect DM predictions, because of their merging at patch boundaries. Compounding annotations as *K*_max_, a technique that, to the best of our knowledge, had not been adopted in cell detection to date, is also found to be key for achieving accurate results without the influence of σ in the DM design.

When predicting DMs with CNNs, the detection threshold is observed to have a substantially different behavior as compared to GT DMs. GT DMs have peaks at 100% threshold and are therefore robust to this parameter upon application of NMS. Meanwhile, predicted CNNs entail inaccuracies in the absolute DM intensities that may lead to unexpectedly inferior detection results if not diligently analyzed. For example, in Fig. 2, only thresholds inside the range [5,40]% produce consistently superior detection results. Removing this threshold hyperparameter without significantly compromising the detection quality (F1 score) is by itself a fundamental advantage of our probabilistic detection approach. Although the parameters in RF can be seen as a mere replacement of the detection threshold, we show that the former can be effectively learned upon the use of diverse classifier configurations (Fig. 3C and table S2), whereas the latter behaves as a hyperparameter requiring either a prior analysis (similar to Fig. 2B) or computationally expensive tuning strategies (e.g., grid search). In addition, taking model uncertainties into account with deep Bayesian methods results in the additional benefit of better calibrated predictions, as evaluated by their lower Brier score and NLL for two distinct cell types: CAR cells and the denser c-kit^+^ cells. In such setting, we show that the classification of cell proposals is similarly effective with a number of different machine learning classifiers, suggesting that predicted DMs already pose a simple classification problem and that the key contribution resides in our proposed pipeline design rather than the specific classifier used. A relevant question for future work is whether the number of features in the selected RF method can speed up the detection process without compromising the results.

Although taking uncertainties into account in the regression CNN improved the calibration of the predictions, including them in the features for the probabilistic classifier did not significantly affect any evaluated metric. This observation may imply that DM values can somehow reflect uncertainty information that is subsequently exploited by the classifier, consistent with the findings in (*52*) that simply taking uncertainty into account during training of CNNs improves calibration of the results, without the need for postprocessing them. However, uncertainties did not seem to capture information about specific unseen structures (Fig. 4) as has been the case in previous reports (*52*, *61*). Whether this is a limitation caused by our dataset, the chosen Bayesian techniques or the use of DM as a proxy for detection is worth investigating in future research. In particular, although not applied herein because of their impracticable computational complexity, deep ensembles that have produced more accurate uncertainties in other tasks (*68*) may shed light on and provide improvements to our Bayesian approximations.

We believe that our probabilistic cell detection framework offers a paradigm shift in analysis tasks. Although the reported throughput and detection metrics already favor the use of the deterministic interpretation of the outputs achieved with Bayes *+* RF as compared to the previously used CIP method (*14*), it is the superior calibration metrics that justify the proposed probabilistic analysis as a superior option to the more common deterministic alternative. In addition, being our methods automatic, computational time has the potential to improve with future hardware and algorithms, whereas the manual workforce required for CIP is not scalable. As we showcase with the numbers reported for bone marrow stroma, a deterministic interpretation of the results does not contemplate mistakes in predictions. Instead, taking output confidences into account in a probabilistic analysis entails a weighting of the contribution from each prediction to the final results by its associated probability, which, in correctly calibrated models, corresponds to their likelihood of being accurate. The application of this analysis for the characterization of the spatial distribution of CAR cells within the bone marrow has confirmed our previous results and corroborates the preferential localization of this cell type in close association to sinusoidal vessels reported in (*14*). However, the revised results presented herein not only provide a higher statistical power and additional confidence estimates but also reveal substantial differences in specific parameters defining spatial distributions of CAR cells. For instance, the probabilistic interpretation indicates that the amount of CAR cells adjacent to sinusoids is 15.42 ± 13.18% and 14.61 ± 9.86% lower than in the deterministic results for the diaphysis and metaphysis, respectively, where we believe the former to be a more accurate estimate for the reasons explained and demonstrated in this work. Notably, compared to the deterministic analysis, the results for the probabilistic one indicate a higher cell density but a lower amount of cells adjacent to vasculature. As confirmed by the spatial analysis results, this observation can be explained by the fact that cells are relatively less abundant in the vicinity of vasculature than hinted by the deterministic results.

In addition to the hypothesis testing techniques already used in (*14*) for taking sample variation into account, we hereby also take into account variability in the cell detection predictions. This latter variability is estimated from the uncertainty implicit to the method (epistemic) and the uncertainty arising from the observations (aleatoric). Thus, together with earlier sample variation, we account for all possible sources of error in our analysis. Therefore, we trust that the proposed cell detection and probabilistic analysis strategies can become instrumental in future studies of spatial distributions.

We further showcase the transferability of our methods to the detection of c-kit^+^ hematopoietic progenitors, a population supporting the lifelong production of blood cells. Thus, our framework holds great potential to uncover unknown spatial affinities between individual stromal and parenchymal components of multicellular tissues. Such an analysis would be essential for the understanding of organ function and pathological states.

The effect of a probabilistic analysis in a segmentation setting (e.g., sinusoids or arteries) will be a relevant question worth investigating in future work. However, the sampling strategy proposed herein for detection coordinates would not be suitable for representing uncertainty for segmentation tasks, i.e., sampling pixels cannot simulate ambiguities, e.g., in extents along different dimensions, morphology, etc. Consequently, novel methods will need to be devised to establish connections between uncertainty maps and a consistent probabilistic analysis.

Although instance segmentation methods can be used for similar analyses (*69*–*71*), detection frameworks have the benefit that manual labeling of coordinates is substantially faster than individual pixels, especially in 3D data. Furthermore, while other detection methods used for the prediction of bounding boxes consist of multistage approaches that require several postprocessing steps (*41*), we use a DM regression approach that, in its standard form, only requires a threshold-based NMS (*42*). With our contributions, such a threshold hyperparameter is further eliminated with two separate supervised models that constitute an end-to-end framework without compromising the detection accuracy. The additional benefit of our method predicting well-calibrated probabilistic cell proposals is shown to offer a more comprehensive analysis enabling confidence intervals for any hypothesis testing, which we believe will be the base of future image-based quantification in different fluorescence microscopy and other imaging datasets.

## METHODS

### Dataset and tiling strategy

We evaluate our detection strategies on bone marrow samples from the dataset presented in (*14*). The Cxcl12-GFP signal is expressed at high intensities by CAR cells and used as input image. The c-kit signal (emitted by an anti–c-kit antibody) of the same dataset is used for the detection of c-kit^+^ cells in the “Transferability of probabilistic cell detection to a different cell type” section in Results. Both CAR cells and c-kit^+^ cells are manually annotated by their central coordinates in seven different samples with the properties in table S3.

Because large fluorescence microscopy volumes cannot fit in the memory of graphics processing units (GPUs) required for fast application of CNNs, we decompose each sample into a number of 3D patches following a previously reported tiling strategy for 2D images (*62*). This method ensures that output patches, which are smaller than input patches upon application of CNNs, can be seamlessly reconstructed. In summary, Gaussian normalization (subtracting the mean and dividing by SD) is first applied to all samples before resizing them to the isotropic resolution of 1 μm per voxel and zero padding with a margin *l*_pad_. Input patches *x* ∈ ℝ^*l*_in_^ are then extracted with an overlap of *l*_overlap_ between them. Output patches *y* ∈ ℝ^*l*_out_^ are generated as GT DMs from the coordinates annotated within *x* as detailed in the next subsection for supervised training of CNNs. *l*_out_ is determined by the pixels subtracted from *l*_in_ by convolution operations within the regression CNN.

A final output *y*_tile_ ∈ ℝ^*l*^out_tile^^ is obtained from *y* depending on the tiling strategy used. The *M*_conv_ strategy uses *y*_tile = *y*_, and *M*_peak_ subtracts a supplementary margin of 4 μm to avoid duplicated predictions in neighboring patches. *l*_overlap_ is calculated such that the resulting *y*_tile_ are adjacent to each other, except at the sample border, where an additional overlap is permitted to ensure completion of the entire sample volume. The sizes used for each of these strategies are reported in table S4. *l*_in_ is selected to create input patches that are as big as possible while fitting in the GPU memory and being divisible at the different downsampling layers by taking into account the pixels lost because of the corresponding convolutional layers. Smaller *l*_in_ results in a higher ratio of borders in the dataset, which can negatively affect the detection results when using *M*_conv_ tiling. However, as shown in Fig. 2C, the proposed *M*_peak_ strategy used in the rest of the experiments is invariant to such border artifacts, and hence, *l*_in_ may only affect the computational speed.

For the training and evaluation of models, 20% of the samples within the complete dataset *D* (table S3) were split as a separate test set *D*_test_ where the different methods were evaluated. Within the remaining samples (*D*\*D*_test_), 80% were randomly selected for inclusion in a training set *D*_train_ used for the training of DM regression models, while the remaining 20% in a validation set *D*_val_ where different hyperparameters of these models were evaluated and tuned. This separation into training and validation from *D*\*D*_test_ was repeated with the same ratio to create a parallel split ${\tilde{D}}_{\text{train}}$ and ${\tilde{D}}_{\text{val}}$, which were used separately in the training of binary classifiers, as explained in the next sections. The data split is identical for the different evaluated models, such that we can compare them in a paired fashion.

### DM generation

Let *C* ∈ ℝ^*N_c_* × 3^ be the annotated GT locations within an image *x*, where *N_c_* is the number of annotations. Let each location be denoted as *c* ∈ ℝ^3^ and *G*_σ_ the Gaussian function be defined as

$$G_{\sigma }(s)=\frac{1}{\sigma \sqrt{2\pi }}\text{exp}\;(-\frac{s^{2}}{2\sigma^{2}})$$

Compounding *G*_σ_ using the *K*_sum_ strategy commonly used in previous works results in a DM *y* with the value at each pixel *k* ∈ ℝ^3^ calculated as

$$y_{k}=\sum_{c}G_{\sigma }(k-c),\;\text{where}\;∥k-c{∥}_{2}\leq l_{g}$$

considering points only within a radius *l_g_* = 16 μm for computational efficiency, because points further away will have an insignificant contribution.

In the *K*_max_ strategy, the different *G*_σ_ associated to each of the coordinates is compounded by their maximum

$$y_{k}=\underset{c}{\text{max}}(\{G_{\sigma }(k-c)\}),\;\text{where}\;∥k-c{∥}_{2}\leq l_{g}$$

Coordinates are localized in the resulting DMs by the application of NMS with a minimum distance between peaks of 4 μm. This value is chosen as the average cell radius, which was also observed in the training data to be smaller than the minimum distance between any GT annotation points.

### Neural networks for DM regression

The UNet model *f*_UNet_ used herein is based on the one proposed in (*28*), which we adapt to 3D by replacing 2D with 3D convolutional layers, with less feature channels to compensate for the capacity increase related to the use of 3D kernels. In addition, we include residual connections (*29*) for each convolutional block. We define a convolutional block with *q* nodes as

$$C_{q}(a)=\text{ReLu}(\text{ReLu}(h_{q}(\text{ReLu}(h_{q}(a))))+a)$$

where ReLu is the rectified linear unit (*72*), *a* is an activation at any level of the CNN, and *h_q_* is a 3D convolutional layer with a 3 × 3 × 3 kernel and *q* channels.

Denoting 3D max pooling layers with 2 × 2 × 2 kernel as MP and upsampling layers with the same kernel as UP, we build UNet composed of the following layers: *C*_16_ → MP → *C*_32_ → MP → *C*_64_ → UP → *C*_32_ → UP → *C*_16_ → *h*_1_. Skip connections are added to connect blocks with the same resolution as described in (*28*). This UNet is trained with L2 loss on predictions $\hat{y}=f_{\text{UNet}(x)}$ as

$$L=\sum_{\left(x_{k},y_{k})\in (x,y\right)}{\left(y_{k}-{\hat{y}}_{k}\right)}^{2}$$

The Bayesian UNet *f*_Bayes_ uses the same underlying architecture as *f*_UNet_. The last layer *h_q_* (as defined above) is changed from *h*_1_ to *h*_2_ to take aleatoric uncertainty *u_a_* into account, which is calculated simultaneously with the DM as $[\hat{y},u_{a}]=f_{\text{Bayes}(x)}$. A ReLu activation is applied to *u_a_* to ensure positive values. The training loss function then becomes

$$L=\sum_{\left(x_{k},y_{k})\in (x,y\right)}\frac{\left(y_{k}-{\hat{y}}_{k}\right)}{2u_{a,k}}+\frac{1}{2}\text{log}\;u_{a,k}$$

In addition, Monte Carlo dropout is used to take into account epistemic uncertainty *u_e_* by randomly (with a uniform probability of 0.2) deleting some of the convolutional layers *h* except the last one (*53*). With the output being stochastic, 50 samples $\left[{\hat{y}}^{t},u_{a}^{t}\right]$ are drawn at inference to calculate the final $\hat{y}$. For this random sampling, *u_a_* is used as the respective detection mean, and *u_e_* is used as the pixel-wise SD across the 50 samples ${\hat{y}}^{t}$. The parameters above follow the implementation details justified in (*61*).

All CNNs in this work are implemented with TensorFlow v2.3 (*73*) and deployed on an NVIDIA GeForce GTX TITAN X GPU with 12 GB of VRAM (video random access memory). A batch size of 4 is used, which is the maximum size that fits the GPU memory available. All models were trained with an Adam optimizer (*74*) with a learning rate of 10^−3^.

As shown in table S5, other design configurations tested resulted in a slightly inferior F1 score compared to our reference UNet implementation. These include the removal of residual units, changing ReLU activations by leaky ReLU (*75*) and using a Rectified Adam (RAdam) (*76*) optimizer instead of Adam. Given the small changes observed between these configurations, we hypothesize that our findings will likely generalize to other UNet configurations. Although a more exhaustive search [e.g., with AutoML (*77*)] could, in principle, produce a higher F1 score in our dataset, this is beyond the focus of our work, which studies probabilistic detections.

### Probabilistic classification of cell proposals

For the application of probabilistic classifiers, $\hat{N_{c}}$ cell proposals $\hat{C}\in {\mathbb{R}}^{\hat{N_{c}}\times 3}$ are generated from DMs by the application of a threshold-free (setting it to 0) NMS. A feature vector, *v_c_*, is then generated by extracting summary statistics for volumes with different sizes (4, 8, 16, and 32 μm^3^) centered on each of the $\hat{c}\in \hat{C}$ for the available predictions (DM, *u_a_*, and *u_e_*). The summary statistics were heuristically selected as follows:

1. Five percentiles for each of the volumes considered, which are taken uniformly in the range from the 1st to the 99th.

2. The ratio of voxels above five different thresholds *t*, selected uniformly in ranges that differ for each of the volumes considered: *t* ∈ [1,1.5] for DM, *t* ∈ [1,10] for *u_a_*, and *t* ∈ [1,0.2] for *u_e_*.

3. The first four statistical moments: mean, SD, skewness, and kurtosis.

Binary classification models *g* are trained on the features *v_c_* to learn whether each proposal corresponds to a cell. Following the dataset notation in the “Dataset and tiling strategy” section, RF classifiers (*g*_RF_) are trained with 128 trees and gini criterion on ${\tilde{D}}_{\text{train}}\cup {\tilde{D}}_{\text{val}}$. The validation set is not used separately because no hyperparameters need to be tuned. MLP classifiers (*g*_MLP_) are designed with four hidden layers (with 50, 50, 20, and 20 nodes, respectively) with ReLu activations. They are trained for 200 epochs with NLL loss and Adam optimizer on ${\tilde{D}}_{\text{train}}$. The epoch with the best accuracy on the validation set ${\tilde{D}}_{\text{val}}$ is selected.

To provide and compare with a classifier alternative that is independent of the handcrafted features *v_c_* used above, we also used a CNN classifier, *g*_CNN_, that acts directly on a 40 × 40 × 40–voxel region surrounding each proposal $\hat{c}$. These regions are concatenated as channels from the three output volumes: DM, *u_a_*, and *u_e_*. Following the notation in the “Neural networks for DM regression” section, *g*_CNN_ is implemented with a number of convolutional blocks *C_q_*(*a*) = ReLu(*h_q_*(ReLu(*h_q_*(*a*)))) and fully connected layers FC*_q_* with *q* channels, each as *C*_32_ → *C*_64_ → *C*_128_ → FC_2048_ → FC_2048_ → FC_1_. Similarly to MLP above, this CNN classifier is also trained on ${\tilde{D}}_{\text{train}}$. We used the Adam optimizer with a learning rate of 10^−4^ and NLL loss for 20 epochs. The epoch with the best accuracy on ${\tilde{D}}_{\text{val}}$ is then selected.

These binary models output values *p* in the range [0,1] that can be interpreted as probabilities. Therefore, we consider as positive predictions those with *p* ≥ 0.5. In the case of baseline threshold-based detections (UNet and Bayes in Fig. 3), the threshold is selected on the validation set *D*_val_ used in the training of the regression CNNs. Hence, we also study the effect of selecting a threshold probability *p* from the validation set ${\tilde{D}}_{\text{val}}$ for the RF binary classifier used. Figure S6 shows that, in this case, RF [denoted as $\text{RF}(p\leftarrow {\tilde{D}}_{\text{val}})$] performs similarly in detection F1 score to the RF used in the rest of this work, which, as explained above, does not use a separate validation set.

*g*_RF_ and *g*_MLP_ classifiers are implemented with scikit-learn v0.23 (*78*), whereas *g*_CNN_ is implemented with TensorFlow v2.3 (*73*).

### Evaluation details

Coordinates from patches are reconstructed in the context of samples by reverting the tiling strategy in the “Dataset and tiling strategy” section before their evaluation. Subsequently, a pairing function λ is defined that matches each GT coordinate *c* to a predicted coordinate $\hat{c}=\lambda (c)$ by optimizing the linear sum assignment of their respective Euclidean distances

$$\underset{\lambda }{\text{min}}\sum_{c\in C}∥c-\lambda (c){∥}_{2}$$

This equation is solved with the Hungarian algorithm (*79*), with some example cases illustrated in fig. S1.

A TP is considered for pairs ∥*c* − λ(*c*)∥_2_ ≤ *t*_match_, where *t*_match_ is a distance threshold. We set *t*_match_ to 4 μm, the average expected cell radius and the same parameter used as the minimum distance separating peaks in NMS (see above in the “DM generation” section). Assignments ∥*c* − λ(*c*)∥_2_ > *t*_match_ produce an FP for the prediction and an FN for the GT. An FN is counted when no prediction is assigned to a GT [λ(*c*) = ∅], and an FP is counted when no GT is assigned to a prediction $[\lambda^{-1}(\hat{c})=\emptyset$]. From this strategy, we calculate the precision, recall, and F1 score as

$$\text{Precision}=\frac{\text{TP}}{\text{TP}+\text{FP}}\prime$$

$$\text{Recall}=\frac{\text{TP}}{\text{TP}+\text{FN}}\prime$$

$$F1\;\text{score}=2\times \frac{\text{Precision}\times \text{Recall}}{\text{Precision}+\text{Recall}}$$

The calibration of the classification models is evaluated by taking into account the probabilities of predicted coordinates $p(\hat{c})$ estimated by the classifiers described in the previous section. Coordinates predicted by deterministic models are assigned $p(\hat{c})=1$ for TP and FP and $p(\hat{c})=p(\lambda (c))=0$ for FN. Although GT coordinates do not strictly have an associated probability, we denote them as such for convenience. Therefore, annotated GT coordinates have *p*(*c*) = 1, and FP are counted as $p(c)=p(\lambda^{-1}(\hat{c}))=0$. Calibration is assessed with the Brier score and NLL as follows

$$\text{Brier score}=\frac{1}{N_{c}}\sum_{c\in C}{\left(p(c)-p(\lambda (c))\right)}^{2}$$

$$\text{NLL}=-\frac{1}{N_{c}}\sum_{c\in C}(p(c)\cdot \text{log}\;(p(\lambda (c)))+(1-p(c))\cdot \text{log}\;(1-p(\lambda (c))))$$

For instance, a method performing 100 predictions with half true and half false and estimating a uniform *p* = 0.4 for all would yield a Brier score of 0.26 and an NLL of 0.71, while estimating *p* = 0.1 would yield a Brier score of 0.41 and an NLL of 1.20, indicating a poorer performance of the latter predictions.

All models were trained for 200 epochs on the training set *D*_train_, and the epoch with the highest F1 score on the validation set *D*_val_ is selected for posterior evaluation on the test set *D*_test_. Results are calculated for each sample in the test set, following a four-fold cross-validation strategy.

### Pipeline for quantification of bone marrow stroma

The quantification of CAR cells described in the “Probabilistic spatial characterization of bone marrow stromal cells with calibrated cell detection” section in Results is performed on an extension of the dataset provided in (*14*), which includes samples that the CIP methods used in the original work could not successfully analyze. Note that manually labeled samples used for the evaluation of the detection methods form a subset of this data. This dataset was also used in (*62*) using CNNs to segment sinusoids, arteries, and a tissue mask, which we use in our current analysis. Sinusoids and arteries are used herein as reference structures to evaluate potential spatial distributions of CAR cells relative to them. The tissue mask defines the voxels inside the specimen where analysis must occur, such that out-of-tissue empty regions are ignored in spatial analysis.

As proposed in (*14*), cellular distributions are analyzed by using CDFs of distances to segmented structures. The ESD aggregates distances from all background voxels to their respective closest foreground voxel (herein sinusoids or arteries), and its CDF describes the proportion of volume left for cells to distribute at different distances. We compare distances from predicted cell locations to a segmented structure using the ESD of the latter in CDF form. This comparison allows for statistical testing of patterns in the localization of cells relative to the ESD. Namely, an attraction pattern is suggested when the CDF of distances from cells is above that of a baseline ESD and conversely an avoidance pattern when it is below.

Analysis is performed on a subset of cells $\tilde{C}$ obtained from the predictions $\hat{C}$ proposed by a trained Bayes *+* RF. In a deterministic analysis, all predictions with probabilities of at least 0.5 are considered

$$\tilde{C}=\{\hat{c}\;|\;p(\hat{c})\geq 0.5,\forall \hat{c}\in \hat{C}\}$$

In probabilistic analyses, coordinates $\hat{C}$ are sampled according to their probabilities

$$\tilde{C}=\{\tilde{c}∼p(\hat{c}),\forall \hat{c}\in \hat{C}\}$$

To incorporate the effect of cell predictions with different probabilities, the above analysis is repeated multiple times (herein *T* = 50), which, according to a Monte Carlo interpretation of simulations (*15*), permits for testing differences with a significance level α = 2/(*T* + 1) = 0.04. Each probabilistic ESD is calculated by random sampling from the original ESD of a number voxels *w* calculated from a Poisson distribution as $w∼\text{Pois}\;(\tilde{N_{c}})$, as described in (*15*). CDF envelopes in this setting are created as in (*14*) by (i) estimating the probability density function of the distances with Gaussian kernels and Scott method (*80*), (ii) evaluating the CDF at the same distance values for all replicates, and (iii) using the maximum and minimum values at each evaluated distance as the upper and lower envelope lines, respectively.

### Statistical tests

The two-sided Wilcoxon signed-rank test, which is nonparametric to avoid any assumption of normal distribution, is used to assess differences between cell detection results in a paired manner between samples. For the study of unpaired data, the Mann-Whitney *U* test is used, which is nonparametric and allows the assessment of statistical differences when the sample sizes of different groups differ. The two-sample Kolmogorov-Smirnov test is used to compare differences in distance distributions when assessing spatial patterns.

### 3D bone microscopy of bone marrow

Methods for 3D imaging and microscopy datasets of BM were previously reported in (*14*). Additional labeling of hematopoietic progenitors was obtained by immunostaining with goat anti-mouse c-kit (R&D Systems).

### Animal studies

Animals were maintained in the animal facility of the Biologisches Zentrallabor, University Hospital Zurich (USZ-BZL) and treated in accordance to guidelines of the Swiss Federal Veterinary Office. Experiments and procedures were approved by the Veterinäramt des Kantons Zurich, Switzerland.

## Supplementary Materials

### This PDF file includes:

Figs. S1 to S6 Tables S1 to S5 Abbreviations
