# Supplementary material for: Probabilistic spatial analysis in quantitative microscopy with uncertainty-aware cell detection using deep Bayesian regression
Source: Sci Adv. 2022 Feb 4;8(5):eabi8295. doi: 10.1126/sciadv.abi8295 (PMC8816343; doi:10.1126/sciadv.abi8295)
Supplement: Supplementary file 1 — Figs. S1 to S6 Tables S1 to S5 Abbreviations [file sciadv.abi8295_sm.pdf]

Science Advances
AAAS

Supplementary Materials for

Probabilistic spatial analysis in quantitative microscopy with uncertainty-aware cell detection using deep Bayesian regression

Alvaro Gomariz*, Tiziano Portenier, César Nombela-Arrieta, Orcun Goksel

*Corresponding author. Email: alvaroeg@vision.ee.ethz.ch

Published 4 February 2022, *Sci. Adv.* **8**, eabi8295 (2022)
DOI: 10.1126/sciadv.abi8295

This PDF file includes:

Figs. S1 to S6
Tables S1 to S5
Abbreviations

Figures

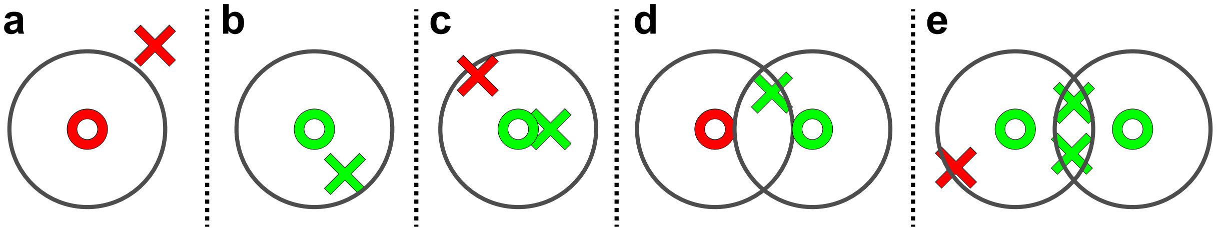

Fig. S1: Illustration of Hungarian matching between predicted (×) and GT (○) coordinates in different scenarios. Every GT annotation (○) is paired as a positive match (TP paired, colored in green) with one and only one prediction point (×) that lies within a predefined (*e.g.* cell) radius, denoted by the gray circles. Negative matches are coloured in red, and denote FN for ○, and FP for ×. (**a**) If predictions are outside the threshold distance of their corresponding GT, they are counted as FN and FP respectively. (**b**) If the distance is below the threshold, both are counted as a single TP. (**c-e**) Multiple potential matches are resolved by minimizing the linear sum assignment, *i.e.* Hungarian matching, *e.g.* (**c**) Only the closest prediction counts as TP, while the others as FP, if multiple are within the distance threshold. (**d**) If the prediction is within the distance threshold to two different GT, only the closest one counts as TP, while the other becomes a FN. (**e**) With multiple predictions within the range of two different GT points, the further one counts as FP, while the other two form TP pairs.

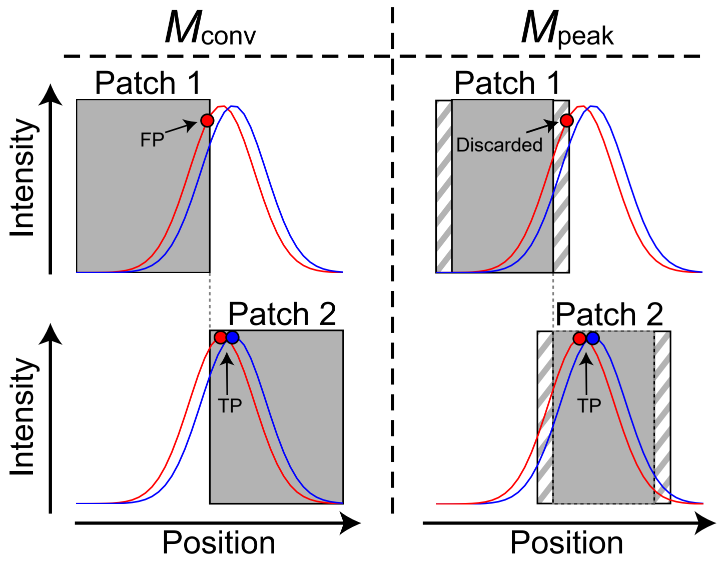

Fig. S2: 1D illustration of the different margin strategies employed. The red and blue curves show sample intensity profiles of the predicted and GT DMs, respectively. The circles show the detection after NMS corresponding to each of the curves. For predictions (red), the circles depend on the selected patch (rows) and the margin strategy (columns). The gray areas indicate the extends of each patch. The gray stripes show the margins discarded in M_{peak} to avoid the FP occurring at the patch border for M_{conv}. Whereas both strategies are capable of detecting the TP, it can be seen that M_{conv} produces an extra FP in a neighbour patch, which is successfully avoided with M_{peak}.

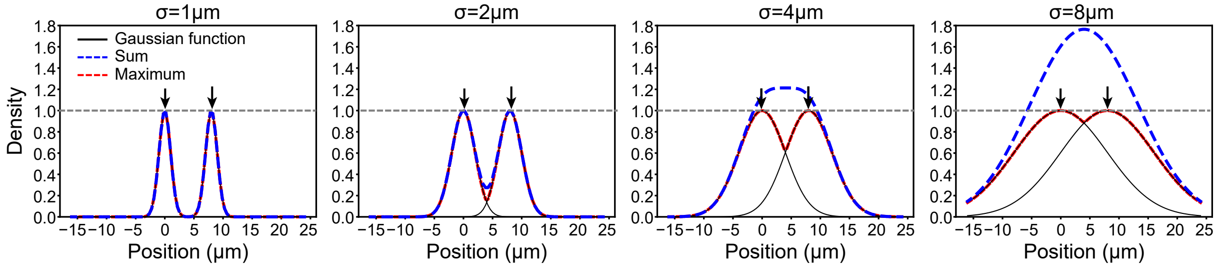

Fig. S3: 1D illustration of the compounding of Gaussian kernels with different kernel sizes σ. Black arrows illustrate the coordinates of two different cells which are one diameter ($8\,\mu\mathrm{m}$) apart. Compounding of kernels by their maximum (K_{max}) allows to discern their respective peaks regardless of the σ value and the resulting density value is bounded (dashed gray line) within an interval, allowing for unified interpretation of a peak and a fixed dynamic range of GT DMs desirable in CNN training. Meanwhile, compounding by their sum (K_{sum}) merges peaks into a single one for values of σ greater than $2\,\mu\mathrm{m}$, and leads to density values higher than those of the original Gaussians, hence confounding peak density with amplitude while hampering peak detection and threshold-based methods.

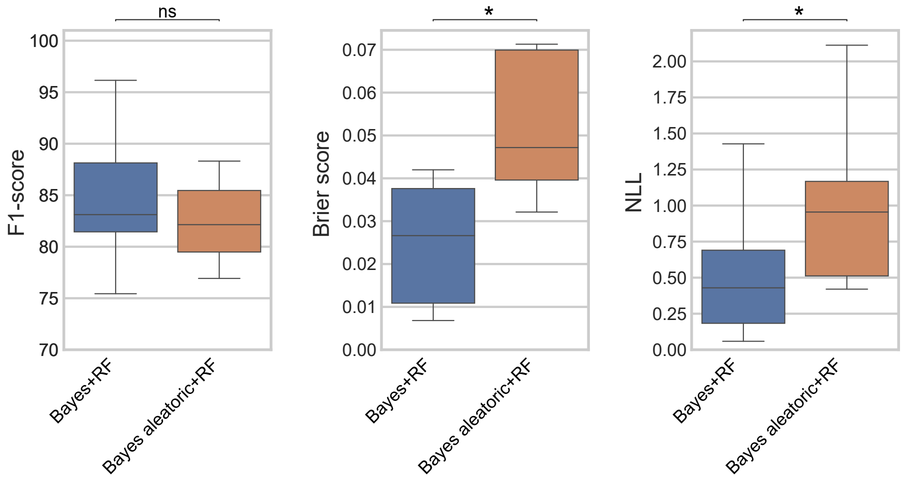

Fig. S4: Comparison of prediction metrics when removing epistemic uncertainty. Our proposed *Bayes+RF* model, which considers both aleatoric and epistemic uncertainties, is compared with a *Bayes aleatoric+RF* alternative that only uses aleatoric uncertainty. The latter avoids additional inference runs for epistemic uncertainty computation, therefore running faster at test time, however with a much reduced probabilistic interpretation of predictions, which is at the focus of our work.

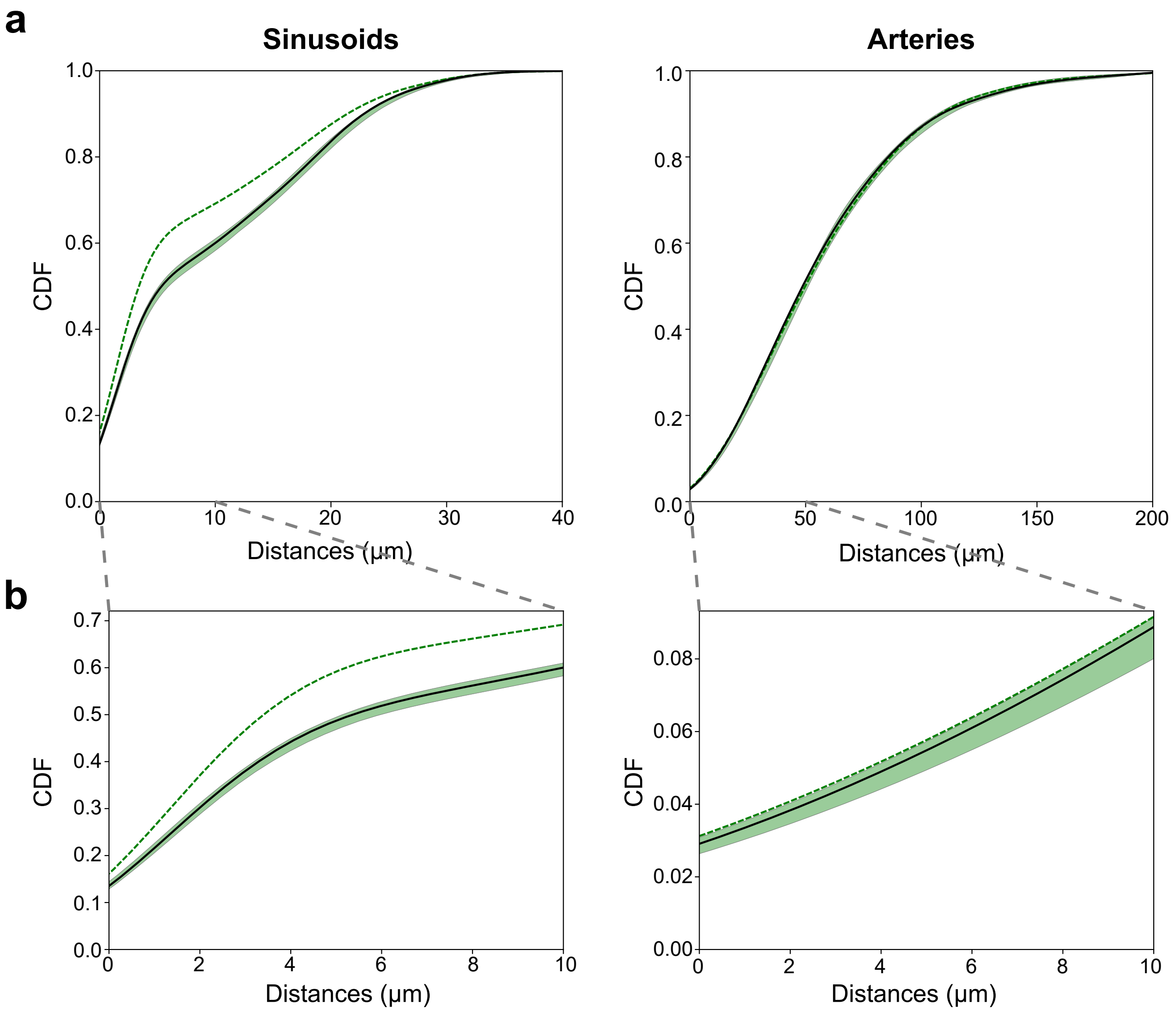

Fig. S5: Comparison of spatial analysis results following our deterministic and probabilistic interpretation with their corresponding GT manual annotations. The same entire sample used for Fig. 7 is employed here, *i.e.* only the GT curve is added in comparison. The analysis is detailed in *Results - Probabilistic spatial characterization of bone marrow stromal cells with calibrated cell detection* and *Methods - Pipeline for quantification of bone marrow stroma*. A black solid line is used for GT results, a green dashed line for those corresponding to deterministic predictions. Envelopes show the results for the probabilistic predictions as the maximum and minimum CDF values for each of the replicates at each distance. (**a**) The results are shown for all possible distances within the sample for sinusoids (left) and arteries (right). (**b**) Zoom-in of (a) for distances from 0 to 10 μm. The results show that the GT analysis results fall within the envelope produced by our deterministic analysis, whereas it differs from the line produced by the deterministic option, especially for sinusoids.

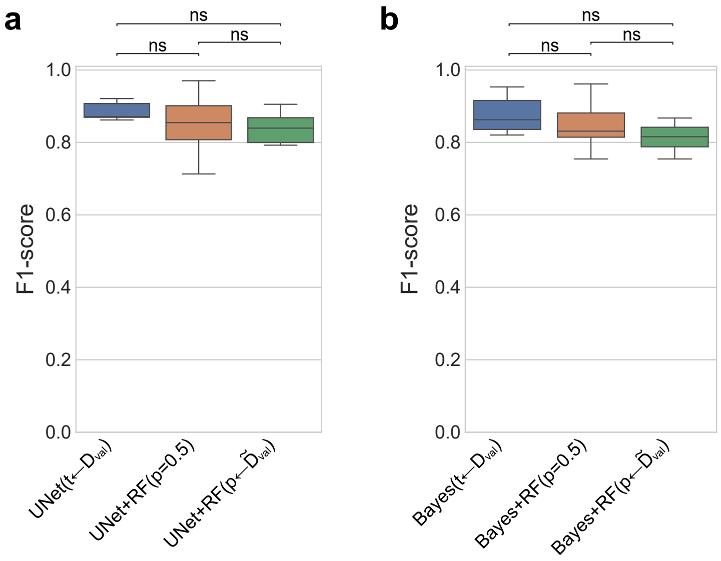

Fig. S6: Comparison of probabilistic models with *RF* using different criteria for thresholding the probability p. Given the probabilistic interpretation, p can be set to 0.5, which is named here "RF(p=0.5)". For comparison with threshold-based detection methods (UNet or Bayes) where this threshold t is selected from the validation set ($t \leftarrow D_{\text{val}}$), we also consider an RF where the probability p is selected from its corresponding validation set as $RF(p \leftarrow \tilde{D}_{\text{val}})$. These results confirm that the detection results are not significantly different across these methods, although the probabilistic interpretation of $p = 0.5$ produces slightly superior results.

Tables

Table S1: Effect of GT DM design on the detection $F1$-score of predicted DMs. The distinct DM design strategies presented in Section *Results - Tiling strategy and density map design for large images* are employed here to generate GT DMs for training *UNet* models. The results are evaluated as described in Section *Results - Probabilistic classification of cell proposals* for *UNet*, namely by selecting the best detection threshold for each model on a separated validation set before evaluation on the test set. $F1$-score (mean±SD) results are shown for each tiling strategy as columns and for each σ value as rows. The results confirm that the strategy selected for the subsequent experiments in this work (i.e., K_{max} & M_{peak} with σ=4 μm) provides the best detection $F1$-score (highlighted in bold). In addition, some similarities with the trends in the results for detection in GT DMs in Fig. 2 are observed. First, K_{max} & M_{conv} leads to slightly lower $F1$-score than K_{max} & M_{peak} for all the evaluated σ values, which can be attributed to the sensitivity of the former strategy to the detection threshold, similarly to what is observed for GT DMs in Fig. 2d. Second, K_{sum} & M_{peak} produces a similar $F1$-score to K_{max} & M_{peak} when σ is 1, 2, or 4 μm (i.e., less than or equal to typical object size being detected), but a substantially lower $F1$-score when σ=8 μm, consistent with the observations for GT DMs in Fig. 2e. Despite sometimes relatively small differences, these results support the analysis in Fig. 2 in that K_{max} & M_{peak} is a safer strategy, especially considering its lower sensitivity to σ – a hyperparameter that has not been analyzed well in similar DM regression methods, as described in the main text.

	K_{max} & M_{peak}	K_{max} & M_{conv}	K_{sum} & M_{peak}
σ=1 μm	86.41±7.13	84.40±7.81	86.09±8.45
σ=2 μm	86.48±5.31	85.54±7.10	87.79±4.84
σ=4 μm	**89.39±3.96**	85.47±6.32	88.39±4.46
σ=8 μm	88.37±5.54	85.91±5.02	76.58±13.46

Table S2: Effect of RF hyperparameters on detection $F1$-score. *# trees* is the number of trees in the forest, *# samples split* is minimum number of samples to split a node, and *# samples leaf* is the minimum number of samples needed for a node to be a leaf. The RF setting employed through the rest of this work is highlighted in bold. These results show that the $F1$-score is largely unaffected by the choice of RF hyperparameters.

# trees	# samples split	# samples leaf	$F1$-score
32	2	1	84.17±6.73
64	2	1	83.60±6.96
**128**	**2**	**1**	**84.84±6.74**
256	2	1	83.97±6.79
128	1	1	84.25±6.80
128	3	1	83.85±6.78
128	4	1	84.61±7.00
128	2	2	84.42±6.64
128	2	3	84.00±7.00
128	2	4	83.75±7.37

Table S3: Summary of labeled dataset employed for the evaluation of detection methods. Note that ckit$^+$ cells are only used in *Results - Transferability of probabilistic cell detection to a different cell type* and CAR cells are employed for all other results.

Sample ID	# patches	# annotated CAR cell coordinates	# annotated ckit$^+$ cell coordinates
1	16	440	873
2	24	549	1230
3	12	171	1143
4	12	332	530
5	16	338	1574
6	16	566	887
7	20	642	1263
TOTAL	116	3038	7500

Table S4: Tiling parameters. The employed parameters are included for the two strategies presented: M_{conv} and M_{peak}. Sizes are in voxels.

**Tiling strategy**	**Input (l_{in})**	**CNN output (l_{out})**	**Output ($l_{\text{out\_tile}}$)**	**Padding (l_{pad})**	**Overlap (l_{overlap})**
M_{conv}	$64 \times 156 \times 156$	$24 \times 116 \times 116$	$24 \times 116 \times 116$	$20 \times 20 \times 20$	$40 \times 40 \times 40$
M_{peak}	$64 \times 156 \times 156$	$24 \times 116 \times 116$	$16 \times 108 \times 108$	$24 \times 24 \times 24$	$48 \times 48 \times 48$

Table S5: Evaluation of different UNet design and optimization settings. *Standard* refers to the UNet model employed through the rest of this work and described in *Methods - Neural Networks for density map regression*, which is seen to achieve a better $F1$-score than the alternatives tested herein. *No residual units* is the more conventional UNet alternative without residual connections. In *Leaky ReLU* all ReLU activations are changed by leaky ReLU. RAdam replaces the Adam optimizer with Rectified Adam.

Model	Standard	No residual units	Leaky ReLU	RAdam
$F1$-score (mean±SD)	89.39±3.96	88.79±4.93	88.97±3.70	87.59±7.33

Abbreviations

- CAR: CXCL12-abundant reticular
- DL: deep learning
- CNN: convolutional neural network
- DM: density map
- GT: ground truth
- NMS: non-maximum suppression
- TP: true positive
- FP: false positive
- FN: false negative
- RF: random forest
- NLL: negative log-likelihood
- MLP: multilayer perceptron
- CDF: cumulative distribution function
- ESD: empty space distance
- CIP: conventional image processing
- SD: standard deviation
